# Participation in activities among people with long-term spinal cord injury in Sweden and the USA – an explorative study using secondary data analysis

**DOI:** 10.1038/s41393-025-01073-1

**Published:** 2025-06-05

**Authors:** Jessica L. Dashner, Ulrica Antepohl

**Affiliations:** 1 Program in Occupational Therapy, Washington University School of Medicine, St. Louis, MO USA; 2 Disability and Community Participation Research Office (DACPRO), St. Louis, MO USA; 3 Department of Rehabilitation Medicine, Region Östergötland and Department of Medical and Health Sciences, Linköping University, Linköping, Sweden

**Keywords:** Quality of life, Spinal cord diseases

## Abstract

**Study design:**

Secondary data analysis.

**Objective:**

To explore differences in participation, secondary health complications, and the use of assistive devices and personal assistance among people with long-term SCI in Sweden and the USA.

**Setting:**

Community dwelling individuals with SCI in Sweden and USA.

**Methods:**

Secondary analysis of data collected via PARTS-Mv3 among individuals living with SCI in Sweden (*n* = 73) and in the USA (*n* = 45). Descriptive analyses provided information regarding the participants, their participation in activities, and secondary health complications, together with the use of assistive devices and personal assistance.

**Results:**

Both samples included more males than females. The mean ages for the Sweden and USA samples were 63.7 and 58, respectively. The mean time since injury was 36.3 years for Sweden and 35.9 for the USA. Perceived health was significantly higher in Sweden (3.80) than in the USA (2.89). The USA sample reported higher occurrence of secondary health complications than Sweden. The amount and type of participation in activities varied between countries, so also the use of assistive devices and personal assistance.

**Conclusions:**

Participation differences were identified when comparing individuals with long-term SCI living in Sweden and in the USA. Further explanatory work is needed to determine whether the differences can be attributed to the varying social and health care systems of the two countries. Understanding how cultural differences influence participation can provide valuable information to determine which system is more likely to positively influence the participation of individuals with long-term SCI.

## Introduction

Today, people are aging with SCI and surviving into their 70 and 80 s, many having lived with SCI for 30–40 years [1, 2]. In Sweden, with a population around 10 million, the prevalence for people living with long-term consequences of an SCI is approximately 600 cases per one million people [3], in contrast to the USA with about 330 million residents, where the prevalence has been estimated to be around 915 cases per one million people [4].

Previously, SCI was considered a relatively static condition, meaning that individuals with an SCI were thought to maintain their functional level for most of the remainder of their lives [5]. More recent research has recognized an accelerated aging process among people with SCI [1, 6–9]. Early research focused on aging with an SCI from a medical perspective, and a wide range of secondary health complications (SHCs) have been described, the majority of which occur with higher frequency among those with longer SCI duration [6, 10, 11]. However, studies of aging with an SCI and its effect on participation in activities have been sparse. Participation is a multifaceted concept and can be described in several ways [12]. For example, the International Classification of Functioning, Disability and Health (ICF) describes participation as the opportunity to be involved in everyday life [13], while Hollingsworth and Gray [14] further expand upon this definition to also include several aspects of the individual’s own evaluation of participation. Research describes that people with SCI in their 40 or 50 s often require increased help with personal care activities such as transfers, bathing/showering, and dressing in their depending on injury level and severity [9, 15]. More recent research [16] shows that aging with an SCI is a complex daily issue, and access to adequate assistive devices and personal assistance is needed/emphasized for people with a long-term SCI, to enable them to continue to participate in desired activities despite the possible experience of SHCs.

Government policies in Sweden and the USA differ in their approach to such support for people with disabilities. In Sweden, laws regulate the right of the person with a disability to receive coverage for assistive devices and personal assistance on a national level [17, 18]. Individuals with disabilities may need to pay a portion out of pocket for assistive devices; however, this amount is only a fraction of the actual cost. The law regulating personal assistance is somewhat complex because, for example, after the individual turns 65, further hours of personal assistance are not granted. This due to a transition from disability-related benefits to more age-related benefits.

The USA system is more complicated, and funding for services can come from many different sources or combinations of services [19, 20]. People with long-term SCI may be covered by Medicare, Medicaid, private insurance, or Veterans Affairs (VA) benefits [21]. Most funding sources for assistive devices only provide a new mobility device every five years or more unless there is a certificate of medical necessity, supported by a physician’s prescription and letters of justification. To be eligible for federal funding for durable medical equipment (e.g., power or manual wheelchairs, scooters, walkers, crutches, or canes) to be used in the home under Medicare Part B (which only covers 80% of the assistive device cost), one must be 65 years or older or have a qualifying disability. Medicare does not cover personal assistance [22]. Low-income individuals may also be eligible for a joint federal-state Medicaid program, which covers the 20% co-pay for durable medical equipment. However, coverage for personal assistance varies from state to state [23, 24]. Private insurance policies also vary by company and can provide coverage prior to qualifying for Medicare or may serve as a supplement for the 20% of the cost not covered by Medicare. In addition, many military veterans use VA benefits to fund both assistive technology and personal assistance [21].

After reviewing the literature, to our knowledge there is a lack of research about the potential factors, such as health and governmental policies support influencing participation among people with long-term SCI. In addition, participation differences among individuals living with long-term SCI in different cultures have not been explored. Therefore, the objective of this descriptive study was to explore differences in participation, SHCs, and the use of assistive devices and personal assistance among people with long-term SCI in Sweden and USA.

## Methods

A secondary analysis was used to address the objective of this study. The process for secondary analysis described by Johnston [25] served as a guide. The main steps in the process were (1) Develop research questions, (2) Identifying datasets, and (3) Evaluating datasets to ensure the appropriateness for the research topic.

### Participants and procedure

Data for the participants in Sweden were extracted from a previous study conducted by the last author [26], and the objective of the study was to describe participation in activities and explore the relationship with SHCs among persons aging with a traumatic SCI. A total of 121 community-dwelling persons registered at the regional SCI outpatient center, among those, a total of 73 answered the survey by phone and thus constitute the Sweden sample.

**Fig. 1:**
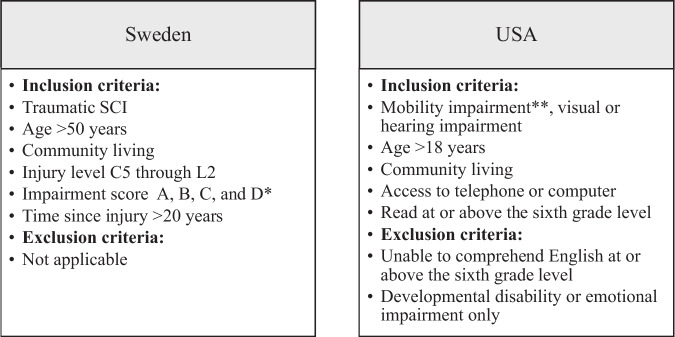
Inclusion- and exclusion criteria for the original datasets used for the secondary data analysis. ^*^According to the ASIA Impairment Scale (ASIA) [31]. ^**^SCI, multiple sclerosis, cerebral palsy, etc.

Data for the USA participants were extracted from a larger national sample with 604 participants (of which 139 had an SCI), who were recruited through several national organizations working for people with disabilities. Data collection was done through a web-based survey. Data from this sample have been used for several articles with different objectives [14, 27] Inclusion and exclusion criteria for the Swedish original study and the larger national sample in the USA are shown in Fig. 1.

To address the objective of this current study, a purposive sampling was used within the USA data, to identify a study sample matched with the Sweden study, based on the following inclusion criteria: (1) men and women with an SCI, (2) aged 50 years or older, and (3) at least 20 years post-injury. This resulted in a USA sample of 45 participants for the current study.

### Survey

The Participation Survey—Mobility version 3 (PARTS-Mv3) is a self-report instrument designed to measure participation of persons with mobility impairments in different activities. An advantage of this instrument is that it captures several subjective dimensions of participation and provides information about what limits participation.

It is divided into two sections; the first one, referred to as the Characteristics of the Respondent (CORE), measures items such as general health on a 5-point Likert scale, with higher scores indicating better health, the prevalence of SHCs, and the general use of assistive devices. The second section measures participation for persons with mobility impairments in 24 activities. For each activity, four components of participation (temporal, evaluative, health-related, and supportive) are measured. Temporal questions focus on frequency of, or time spent participating in an activity. Evaluative questions focus on choice, control, satisfaction, and importance of participating in a particular activity. Health-related questions focus on whether the activity is limited by for example pain and/or fatigue. Supportive questions address the personal and/or assistive devices that the person may use to participate. The original PARTS has been tested as a reliable measure with moderate-to-high internal consistency and stability [28]. In the USA version of the survey, the University’s Institution Review Board requested a response option for individuals who preferred not to answer the questions. In the Sweden version, individuals only answered the questions for the activities in which they participated.

### Data analysis

To answer the objective for the study, the researchers decided to use the temporal, supportive, and the health-related questions for 10 of the 24 PARTS activities. The selection of the 10 activities was based on the findings in an earlier study by the last author [16]. Data analyses were conducted using the IBM Statistical Package for Social Sciences (SPSS) statistics, version 22.0 (Armonk, NY: SPSS, Inc.).

To guide the data analysis, the following questions were used: (1) Do differences in general health and/or SHCs exist between persons with a long-term SCI in Sweden and the USA? (2) Are there differences in the use of assistive devices and personal assistants between persons with a long-term SCI in Sweden and the USA? (3) Are there differences in participation in activities between persons with a long-term SCI in Sweden and the USA? Descriptive statistical analyses (e.g., mean, standard deviation, and frequencies) were performed to explore and describe the data and the study population. To examine differences between factors such as age, time since injury, SHCs, and participation in activities, the independent *t*-test, crosstabs, and *X*^2^ test were used where appropriate to compare the samples. The significance level was set to *P* < 0.05.

### Ethical considerations

This study expands on two separate studies that were conducted with approval from the Human Ethics committee in Stockholm, Sweden (no: 2014/214-31/3) and the University’s Institution Review Board, USA (no: 201101785), and where informed consent was obtained from the participants. We certify that all applicable institutional and governmental regulations concerning ethics were followed during course of this research.

## Results

### Demographic data

Demographic data for the participants with SCI, and the use of mobility devices are shown in Table 1. Significant differences in age were found between the sample in Sweden and that of the USA (*P* > 0.001), whereas no significant differences were found regarding time since injury. The levels of injury for the Sweden sample were distributed as follows: C5-C8 (*n* = 27; 37.0%), T1–T6 (*n* = 14; 19.2%), T7–T12 (*n* = 24; 32.9%), and L1–L2 (*n* = 8; 11.0%). In addition, the severity of SCI was distributed as follows: A (*n* = 41; 56.2%), B (*n* = 5; 6.8%), C (*n* = 12; 16.4%), and D (*n* = 15; 20.5%). Details regarding level and severity of injury were not reported for the USA sample since it was not included in the origin study in USA.

**Table 1:** Demographics of the study population.

	Sweden (*n* = 73)	USA (*n* = 45)
Gender n (%)		
Men	55 (75.3)	32 (71.1)
Women	18 (24.7)	13 (28.9)
Age		
Mean	63.7 ± 9.4^a^	58.0 ± 5.5
Range	50–87	50–71
Time since injury		
Mean	36.3 ± 9.2	35.9 ± 8.7
Range	20–55	20–59
Educational status n (%)		
High school or less	29 (39.3)	6 (13.3)
University/college	44 (60.3)	39 (86.7)
Marital Status n (%)		
Married	33 (45.2)	26 (57.8)
Divorced	4 (5.5)	13 (28.9)
Widowed	3 (4.1)	1 (2.2)
Living Alone	26 (35.6)	9 (20)
Member unmarried couple	7 (9.6)	3 (6.7)

^a^mean ± standard deviation.

### Health and secondary health complications

The mean score for perceived general health reported on the CORE (5-point Likert scale) in the Sweden sample was 3.8 ± 1.04, and the USA sample was 2.89 ± 0.80. There were significant differences (*P* = 0.05) between the samples regarding participants perceived general health, including significant differences between the groups for the prevalence of SHCs (Table 2). The USA sample reported a higher occurrence of all SHCs except for poor balance/falls. Significant differences were found for spasticity, neck/arm problems, upper respiratory infections, high blood pressure, depression, urinary tract infections, bladder incontinence, bowel incontinence, weight problems, and skin problems. Pain and fatigue were highly reported in both samples, but no significant differences in occurrence of pain and fatigue between the two groups.

**Table 2:** Participants reporting secondary health complications.

	Sweden n (%)	USA n (%)
Spasticity^***^	9 (12.3%)	26 (57.8%)
Neck/Arm Problems^***^	13 (17.8%)	22 (48.9%)
Upper Respiratory Infections^*^	1 (1.4%)	4 (8.9%)
High Blood Pressure^**^	4 (5.5%)	11 (24.4%)
Depression^**^	1 (1.4%)	7 (15.6%)
Urinary Tract Infection^***^	3 (4.1%)	13 (28.9%)
Bladder Incontinence^***^	8 (11.0%)	18 (40%)
Bowel Incontinence^***^	7 (9.6%)	18 (40%)
Weight Problems^***^	0 (0.0%)	9 (20%)
Skin Problems^*^	9 (12.3%)	13 (28.9%)
Poor Balance/Falls	7 (9.6%)	5 (11.1%)
Fatigue	52 (71.2%)	38 (84.4%)
Pain	55 (75.3%)	37 (82.2%)

^*^P < 0.05, ^**^P < 0.01, ^***^P < 0.001.

### Participation in activities

The frequency of participation for each activity is shown in Table 3. Significant differences were found for dressing, bathing, and leisure activities, indicating that the Sweden sample spent more time in these activities. In contrast, the USA sample spent significantly more time working inside, leaving home, and exercising.

**Table 3:** Frequency of participation in activities.

	Sweden (*n* = 73)	USA (*n* = 45)
^1^Dressing^**^	2.90 ± 0.93^a^	2.76 ± 1.15
^1^Bathing^**^	3.34 ± 0.75	2.67 ± 0.98
^1^Meals	2.89 ± 1.30	3.07 ± 1.18
^2^Work inside^*^	2.55 ± 1.38	3.75 ± 0.59 (40)^b^
^3^Leaving home^*^	3.30 ± 0.91	3.84 ± 0.37
^4^Exercise^**^	2.49 ± 1.37	2.95 ± 1.25 (43)
^4^Active recreation	1.89 ± 1.25	1.76 ± 1.13 (34)
^4^Leisure^*^	3.99 ± 0.12	3.73 ± 0.63 (41)
^5^Socializing	3.34 ± 0.99	2.98 ± 0.90 (42)
^6^Employment	1.53 ± 0.84	2.00 ± 0.91 (37)

^*^P < 0.05; ^**^P < 0.01.

^1^Scale: 1 = < 10 min; 2 = 11-20 min; 3 = 21-30 min; 4 = > 40 min.

^2^Scale: 1 =  never/rarely; 2 = 1-2x/week; 3 = 3-4x/week; 4 = >5x/week.

^3^Scale: 1 = never/rarely; 2 = 1-2x/month; 3 = 1-2x/week; 4=daily/almost daily.

^4^Scale: 1 = never/rarely; 2 = 1-2x/month; 3 = 1-2x/week; 4 = >2x/week.

^5^Scale: 1 = never/rarely; 2 = 1-2x/week; 3 = 3-4x/week; 4 = daily/almost daily.

^6^Scale: 1 = none; 2 = 1–20 h/week; 3 = 21-40 h/week; 4 = more than 40 h/week.

^a^Mean ± SD.

^b^Actual response rate for the activity, due to the request from the University’s Institution Review Board for a response option “*I prefer not to answer*”.

**Fig. 2:**
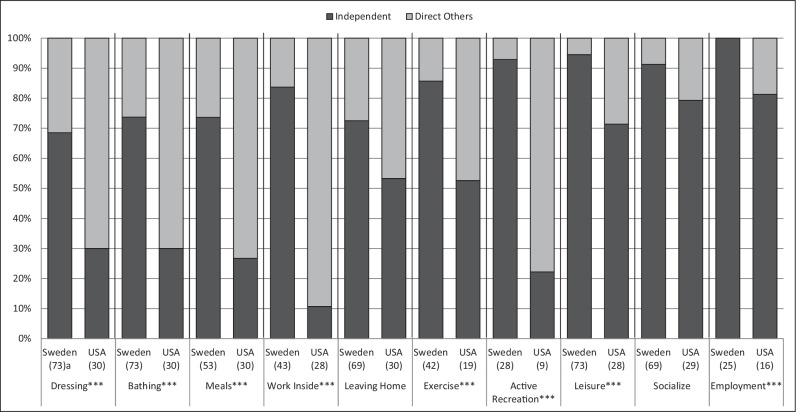
Type of participation: Independent or Direct Others to Assist. ^***^P < = 0.001. a For Sweden, *n* = represents the participants who actually participated in the activity. b For USA, *n* = Actual response rate for the activity, due to the request from the University’s Institution Review Board for a response option “I prefer not to answer”.

Participation in activities can be done either independently or by directing others to help; Fig. 2 shows the distribution between these two options. Overall, participants in the Sweden sample reported that they participated independently in every activity to a higher extent than the participants in the USA sample. Significant differences were found for each activity except for leaving home and socializing.

Pain and fatigue were the most frequently reported SHCs in both samples. Looking at them in relation to participation, significant differences were found for pain between the samples, indicating that participants in the USA sample were more likely to experience pain when participating in exercise (*n* = 25; 75.8% *P* = 0.05), active recreation (*n* = 13; 86.7% *P* = 0.05), leisure (*n* = 30; 69.8% *P* = 0.01), and socializing (*n* = 30; 69.8% *P* = 0.01). In addition, significant differences were found for fatigue between the samples. Participants in the USA sample were more likely to experience fatigue when participating in bathing (*n* = 24; 53.3% *P* = 0.05), meal preparation (*n* = 22; 48.9% *P* = 0.01), working inside (*n* = 28; 66.7% *P* = 0.01), exercise (*n* = 25; 75.8% *P* = 0.001), active recreation (*n* = 10; 66.7%, *P* = 0.05), leisure (*n* = 28; 65.1% *P* = 0.001), and employment (*n* = 16; 69.6%, *P* = 0.05).

### Personal assistance and assistive devices

Table 4 further expands on the result in Fig. 2 and shows how the participants in the samples used paid and unpaid help when directing others to assist them in participating in activities. Participants from both samples used assistance from others in every activity category except for employment, wherein the Sweden sample utilized no personal assistance. Significant differences were found for help received with dressing, bathing, working inside, and socializing.

**Table 4:** Unpaid and paid personal assistance used in activities.

	Sweden	USA
^**1**^**Dressing**	*n* = 23^a^	*n* = 21^b^
Unpaid^**^	2.22 ± 1.31^c^	2.62 ± 0.92
Paid^*^	3.09 ± 1.35	3.29 ± 1.06
**Bathing**	*n* = 19	*n* = 21
Unpaid^*^	2.16 ± 1.34	2.57 ± 1.78
Paid	3.00 ± 1.33	3.05 ± 1.28
**Meals**	*n* = 14	*n* = 22
Unpaid	1.93 ± 1.14	2.82 ± 1.01
Paid	3.21 ± 1.05	2.64 ± 1.37
**Work inside**	*n* = 7	*n* = 25
Unpaid^*^	2.29 ± 1.38	2.76 ± 0.88
Paid	3.14 ± 1.46	2.92 ± 1.04
**Leaving home**	*n* = 20	*n* = 14
Unpaid	2.10 ± 1.25	3.00 ± 1.04
Paid	3.05 ± 1.32	2.79 ± 1.37
**Exercise**	*n* = 6	*n* = 9
Unpaid	2.00 ± 1.27	2.56 ± 1.13
Paid	2.83 ± 1.17	3.00 ± 1.23
**Active recreation**	*n* = 2	*n* = 7
Unpaid	2.50 ± 2.12	2.86 ± 1.07
Paid	2.50 ± 2.12	2.14 ± 1.35
**Leisure**	*n* = 4	*n* = 8
Unpaid	2.25 ± 1.50	3.25 ± 0.89
Paid	3.75 ± 0.50	1.88 ± 1.13
**Socializing**	*n* = 6	*n* = 6
Unpaid	2.67 ± 1.37	3.17 ± 0.75
Paid^***^	2.50 ± 1.64	3.17 ± 0.75
**Employment**	*n* = 0	*n* = 3
Unpaid	Not used	3.00 ± 1.00
Paid	Not used	3.67 ± 0.58

^*^<0.05, ^**^<0.01, ^***^<0.001.

^1^Scale: 1 = Never, 2 = Rarely, 3 = Often, 4 = Always.

^a^Represents all the participants who directed others to be able to participate in the activity in.

^b^Represents actual response rates of the participants for who directed others to be able to participate in the activity in Fig. 1.

^c^Mean ± SD.

Overall, the USA sample used more assistive devices (e.g., shower chairs, reachers, and splints) than the Sweden sample when participating in activities. Significant differences (*P* = 0.001) were found in dressing, working inside, leisure, socializing, and active recreation (*P* < 0.01). However, there was one exception; the Sweden sample reported using more assistive devices for bathing (*P* = 0.001) than the USA sample.

## Discussion

The results in this study describe differences in participation, SHCs, and the use of assistive devices and personal assistance among people with long-term SCI in Sweden and USA. The SHCs reported by the two samples are similar to those reported in previous studies [1, 6–11, 26]. Individuals with SCI from Sweden indicated overall greater perceived health than participants from the USA. This could be explained by the USA sample reporting a higher incidence of SHCs or higher level and severity of injury. It may also be possible that individuals with SCI in the USA have less access to health care services due to barriers in the physical environment, increased individual costs, and policy/funding limitations [29], and therefore increasing the occurrence of SHCs. Further work is needed to understand the relationship to health care access for both samples and develop adequate culturally relevant lifelong support to minimize the aging effect and the consequences of SHCs.

Additionally, the Sweden sample reported spending more time participating in activities. This could be due to the fact, that they reported using less personal assistance and fewer assistive devices when participating in activities. Despite having easier access to assistive devices and personal assistance in Sweden due to the Government policies [17, 18], individuals in the USA sample reported using both more frequently. Differences in the use of assistive devices could indicate differences in the level and severity of injury between the samples or another explanation could be the access to different mobility devices types in each country. For example, the USA sample reporting using more powered wheelchairs, whereas participants in the Sweden sample with higher level injuries were more likely to use powered assisted wheels (e.g. E-motion wheels) on their lightweight wheelchairs. Further research is needed to determine how out-of-pocket payment or more restrictions in the person’s ability to obtain assistive devices affect the match between the technology and the individual’s specific needs to complete a task and/or participate in activities.

When looking more closely at specific activities, there are some interesting differences between the samples. For example, during bathing, individuals from Sweden reported mainly performing the activity on their own, spending more time, and using more assistive devices. This could be explained by cultural differences and the varying value of independence across cultures. Still evident in both samples is a low rate of participation in exercise and active recreation, something that is important to address in the prevention of SHCs. This emphasizes the need for life-long follow-ups with multi-professional teams, something that is in line with earlier studies from Lundström et al. and Molton et al. [26, 30].

### Limitations

The findings from this descriptive study should be interpreted based on the following methodological considerations. First, ethnicity of the participants was not addressed in this study since that is not common research practice in Sweden, something that possible can limit the description of different aspects of participation. Secondly, we were unable to group the participants in the USA sample by level and severity of SCI, which limits the ability to fully compare the two samples. Thirdly, the request from the University’s Institution Review Board for a response option “I prefer not to answer” for the USA study caused some limitations in data analysis. Therefore, generalization of our findings may be limited. At the same time, the present study is exploratory in nature, and our findings can serve as a base for designing further, more explanatory research. Despite these limitations, the results give an insight in characteristics regarding participation, SHCs, and the use of assistive devices and personal assistance among people with long-term SCI among different cultures. Thus, this study can be seen as a pilot study for future research in this important area.

## Conclusion

This study identified several differences in participation, SHCs, and the use of assistive devices and personal assistance for individuals with-long term SCI living in Sweden in the USA. Both countries have very different governmental policies regarding access to services for people with disabilities. Future work is needed to identify, and explain the specific reasons for the participation differences to determine whether one system is more likely to improve participation over another. Comparative studies between countries are important and can provide information to enhance participation in activities for people with a long-term SCI. This is especially true when comparing countries that have such different models of service delivery to further understand the effects of each method on minimizing the aging effect and the consequences of SHC while enhancing participation in activities.

## Data Availability

The datasets generated and/or analyzed during the current study are available from the corresponding author on reasonable request.
